# Removal Mechanism of Oxide Layer on the Surface of Sn-0.4Ti Alloy for Quartz Glass Sealing

**DOI:** 10.3390/ma13112620

**Published:** 2020-06-09

**Authors:** Wanli Hao, Fangzi Li, Yongbo Ma, Weiguang Zhang, Liqun Shi

**Affiliations:** 1 Institute of Nuclear Physics and Chemistry, China Academy of Engineering Physics, Mianyang 621900, China; mianli1976@163.com (W.H.); lfazh2000@sina.com (F.L.); mybmail@163.com (Y.M.); 18981172699@189.cn (W.Z.); 2 Institute of Modern Physics, Fudan University, Shanghai 200433, China

**Keywords:** removal mechanism, oxide layer, Sn-0.4Ti alloy, quartz glass

## Abstract

The oxide layer on the surface of Sn-0.4Ti alloy and its removal mechanism were investigated by coalitional analyses, using XPS and TEM technologies. The results show that a compact SnO_1.65_ oxide layer of less than 4 nm in thickness exists on the surface of Sn-0.4Ti alloy. A large number of TiO_2_ nanoparticles with scale of several to tens of nanometers were grown in Sn-0.4Ti matrix by depleting SnO_1.65_ while welding at 800 °C. These nanoparticles were adhered to the interfacial layer between Sn-0.4Ti alloy and quartz glass, which was formed by the reaction of Sn-0.4Ti and SiO_2_ after SnO_1.65_ removal from the Sn-0.4Ti. This work may promote further works on Sn-Ti design to further improve the welding quality between Sn-Ti alloy and quartz glass, and also provide a feasible research idea to remove the oxide layer on the surfaces of solders.

## 1. Introduction

Glass-metal sealing is widely used in the fields of light source, electric vacuum device and solar receiver tube [1]. The matching sealing between some kinds of glasses, such as borosilicate glass, devitrified glass and Kovar alloy (or stainless steel), has been widely studied [1,2,3,4,5,6]. The sealing between the quartz glass and metal is challenging, because the thermal expansion coefficient of quartz glass is about one order of magnitude lower than that of metals. In a high intensity discharge lamp, the quartz glass tube needs to link with the metal electrode [7]. At present, transition glass sealing and molybdenum foil sealing are common sealing methods, but these methods show some defects, such as low sealing strength, poor hermetic sealing, etc. [7,8]. As far as we know, there is no literature about solders used to directly weld quartz glass and metal. Obviously, using solder to weld quartz glass and metal is a simple process, but the solder must stick to quartz glass, and the welding stress cannot break this quartz glass. We have shown that the Sn-Ti alloy with a Ti content of less than 6% can react with quartz glass at welding temperature to form a compact interfacial layer [9]. Studies on Sn-Ti alloy have been widely reported [10,11,12,13,14,15,16,17,18,19,20,21,22], and Sn-Ti alloy has been used to weld sapphire, diamond grits, zirconia or Poly-Crystalline CVD Diamond Plates in recent years [18,19,20,21,22]. However, almost no reports have focused on Sn-Ti alloy for quartz glass-metal welding except for our previous work [9]. Sn-Ti alloy with low Ti content may have many excellent properties, such as low tensile strength (~13 MPa), low melting point (~505 K) and good plasticity [23]. All of these properties are beneficial to improve the stability of the welding structure between quartz glass and metal. Reducing the strength of the Sn-Ti alloy by adjusting the Ti content, the welding stress can be reduced to a safe value. It is suggested that Sn-Ti alloy is an excellent active solder for quartz glass sealing. However, Sn-Ti alloy with low Ti content is mainly composed of a pure Sn phase [23]. 

A pure Sn surface can generate a compact oxide layer with a thickness of 2 nm in a week [24]. Thus, a Sn-Ti alloy with a low Ti content can also generate a compact oxide layer, which may dramatically reduce the solder activity. It is interesting to note that the Ti in the alloy may remove the surface oxide layer and promote a reaction between our Sn-0.4Ti alloy and quartz glass. However, the detailed removal mechanism of the surface oxide layer has not been clarified.

The present work aims to investigate the removal mechanism of the surface oxide layer of Sn-0.4Ti alloy by analyzing the oxide layer and the interfacial state between the Sn-0.4Ti alloy and the quartz glass using XPS and TEM technologies.

## 2. Materials and Methods 

Sn-0.4Ti alloy was fabricated by smelting pure Sn and Ti metal, and then formed into slices with a thickness of about 0.2 mm by a rolling process. Considering the actual need of engineering applications for solder, this Sn-0.4Ti sample was kept in a vacuum chamber of 50–100 Pa for one year before welding and testing.

XPS analyses were carried out by an ESCALAB 250Xi X-ray photoelectron spectrometer (XPS) (Thermo fisher scientific, Waltham, MA, USA). The sample was a square Sn-Ti slice with size of 15 mm. Monochromatic diffraction of the Al target was used with a beam spot of Φ 0.5 mm in the measurement. At first, the sample surface with no Ar ion sputtering was analyzed, then sputtered for 130 s by Ar ions. Finally, the sputtered surface was analyzed again.

The welding sample for the Sn-0.4Ti alloy and quartz glass was made in the vacuum furnace (Beijing Nanopa Technology Center, Beijing, China) at 800 °C under a pressure of <5 × 10^−3^ Pa. The welding process lasted 10 min. The welded sample was cut by a diamond wheel, then grinded and polished with a 1 μm diamond paste. The interfacial structure of the sample was analyzed using a JEOL JEM-2010 UHR (Tokyo, Japan) transmission electron microscope (TEM) operated at 300 kV. Specimens for TEM observation were processed to a thickness of about 100 nm using a focused ion beam (FIB) with a microsampling system (Hitachi FE-2100, Tokyo, Japan) using a Ga ion beam.

## 3. Results

**Figure 1:**
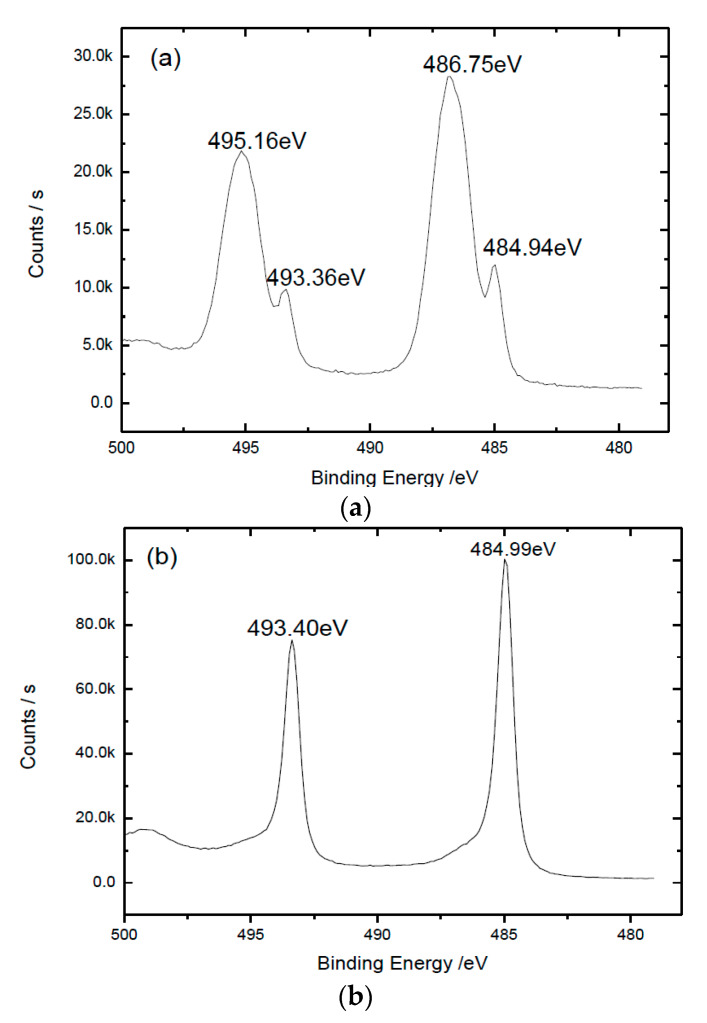
XPS spectrum of Sn 3d2/5 electrons on Sn-0.4Ti alloy (**a**) without sputtering; (**b**) with sputtering for 130 s.

The binding energy of Sn on the surface of the Sn-0.4Ti sample was investigated by X-ray photoelectron spectrometry (XPS). Figure 1a shows that before Ar ion sputtering, Sn 3d2/5 electrons on the sample surface have four binding energies: 484.94 eV, 486.75 eV, 493.36 eV and 495.16 eV. But only the binding energies of 484.9 eV and 493.40 eV were retained after Ar ion sputtering for 130 s, as shown in Figure 1b. By comparing the NIST X-ray Photoelectron Spectroscopy Database [25], both binding energies of 484.9 eV and 493.40 eV belong to the Sn element in atomic state, and the other two binding energies, 486.75 eV and 495.16 eV, belong to the Sn element in the SnO_1.65_ oxide, indicating that the SnO_1.65_ layer was eliminated completely by Ar ions sputtering for 130 s. It is well known that pure Sn can generate a dense oxide layer in atmosphere. Our previous work shows that the main phase of this Sn-0.4Ti alloy is pure Sn. Obviously, although the sample was kept in a vacuum of 50~100 Pa, a compact thin SnO_1.65_ oxide layer was formed on the surface of our Sn-0.4Ti alloy. The fitting on the spectrum further indicates that in all Sn elements detected on the surface of the Sn-0.4Ti alloy without Ar ions sputtering, the Sn in pure Sn phase is about 12%, and in SnO_1.65_ oxide, it is about 88%. As the detecting depths of XPS in inorganic oxides and in metals are about 1.5–4 nm and 0.5–2 nm, respectively, from the detected Sn atom coming from under the oxide layer, the thickness of SnO_1.65_ oxide layer should be less than the XPS detecting range of 4 nm in oxide.

**Figure 2:**
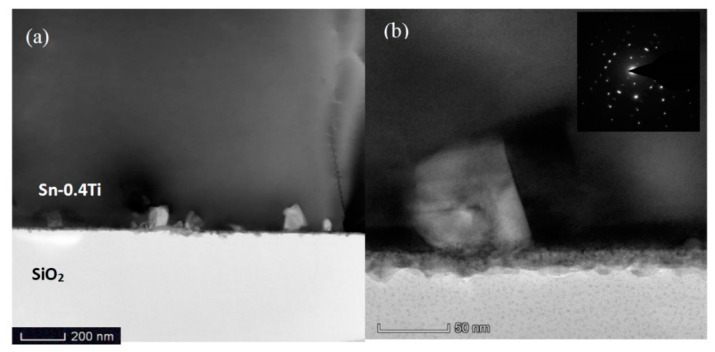
(**a**,**b**) TEM images of the interfacial area between Sn-0.4Ti alloy and quartz glass (the insert shows selected area diffraction (SAED) pattern of the TiO_2_ nanocrystal).

TEM images of the interfacial area between Sn-0.4Ti alloy and quartz glass are shown in Figure 2. In Figure 2a a large number of nanoparticles with a scale of several to tens of nanometers were located in the Sn-0.4Ti matrix. They were connected with the interface layer between the Sn-0.4Ti alloy and quartz glass, which is clearly seen in Figure 2b by a zoomed area containing a single nanoparticle. TEM results show that the thickness of continuous dense interfacial layer is about 20 nm. Some nanoparticles looking like squares are also observed. The side length of the square is up to ~60 nm. 

**Figure 3:**
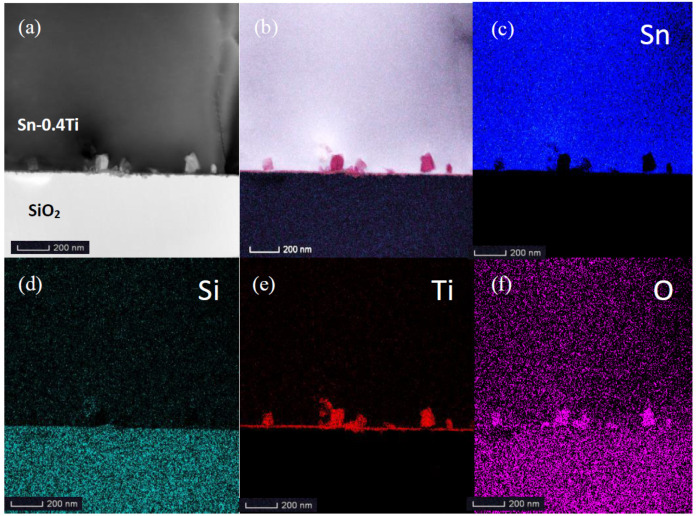
(**a**–**f**) EDS mapping of the interface area between Sn-0.4Ti alloy and quartz glass (color online).

Figure 3a,b represent the analyzed area and synthetic EDS mapping of all elements. The four possible elements contained in the nanoparticles, Sn, Ti, Si and O, are all scanned and shown in Figure 3c–f. Only Ti and O elements with the atomic ratio of about 1:2 are found in the nanoparticles, indicating that these nanoparticles exist in the form of TiO_2_. As with the selected area electron diffraction (SAED) pattern shown in the insert of Figure 2b, no indication of a diffuse ring, but rather, bright diffraction spots, are observed, suggesting that TiO_2_ should crystalize in nanoparticles.

In Figure 3e, a thin TiO_2_ layer between the nanoparticles and Sn-0.4Ti alloy is clearly shown, as reported in the literature [9].

## 4. Discussion

As no TiO_2_ phase exists in the as-received Sn-0.4Ti alloy, the forming mechanisms on TiO_2_ nanocrystals adhering to the interfacial layer of the TiO_2_ nanolayer on the SiO_2_ are put forward. According to our theoretical design of Sn-0.4Ti alloy for quartz glass sealing, the key role of Ti in the alloy is to remove the oxide layer on the alloy surface by its much higher chemical activity than Sn. A displacement reaction between SnO_1.65_ and Ti is shown in Equation (1). The thermodynamic calculation shows that the reaction enthalpy (Δ_f_H) and the reaction free energy (Δ_f_G) are −315.6 kJ/mol and −317.6 kJ/mol, respectively, at 25 °C, which clearly means that this reaction is exothermic and spontaneous. Thus, under the welding temperature of 800 °C, Sn-0.4Ti alloy melts entirely, and Ti atoms diffuse to the surface of oxide layer and react with SnO1.65 according to Equation (1) to generate free TiO_2_ molecules and Sn atoms.

Sn atoms will solute in the liquid alloy, while the TiO_2_ molecules will aggregate into nucleus, growing into nanocrystals in the near surface of liquid Sn-0.4Ti as TiO_2_ is insoluble in liquid Sn-0.4Ti, and the melting point of TiO_2_ (1850 °C) is far higher than the welding temperature (800 °C). The final size of TiO_2_ nanocrystals is related to the density of crystal nucleus and the layer thickness of SnO_1.65_ eliminated by the reaction.

Ti (L) + SnO_1.65_(S) = 0.825TiO_2_ (S) + Sn (L) (1)

**Figure 4:**
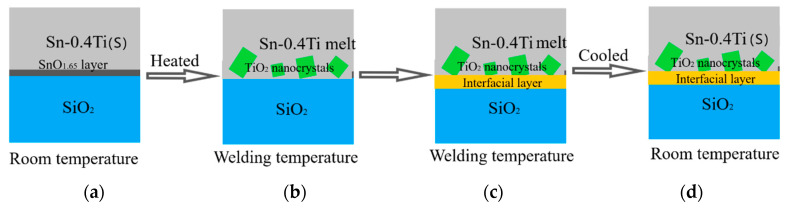
(**a**–**d**) Schematic diagram of the removed mechanism of SnO_1.65_ layer.

In Figure 4, based on the above analysis, the removal process of the SnO_1.65_ oxide layer on the surface of Sn-0.4Ti alloy, and the formation of the interface layer, are shown. At room temperature (25 °C), Ti atoms in alloy cannot diffuse out and react with SnO_1.65_, thus the oxide layer is stable, as shown in (a). When the temperature rises to 800 °C, TiO_2_ nanocrystal particles of several to tens of nanometers in size form in the Sn-0.4Ti near the interface, but cannot cover the SiO_2_, as their dimension is much larger than the thickness of the SnO_1.65_ layer (b). After the SnO_1.65_ layer is eliminated completely by Equation (1), fresh Sn-0.4Ti in fusing state could directly contact with quartz glass. Thus Ti atoms diffuse into the SiO_2_ solid and react with O to generate TiO_2_, which finally grow into a thin nano-layered-structure interface (c,d) connecting TiO_2_ nanocrystals and SiO_2_, as reported in our previous work [9].

## 5. Conclusions

In summary, the removal mechanism of a SnO_1.65_ oxide layer was studied in this paper. A compact SnO_1.65_ oxide layer with a thickness of less than 4 nm was grown on the surface of the Sn-0.4Ti alloy when it was stored in vacuum of 50~100 Pa for a year. The removal of the SnO_1.65_ layer is through the reaction between the diffusing Ti atoms from the liquid Sn-0.4Ti alloy and the O atoms of SnO_1.65_ at our welding temperature of 800 °C. The produced TiO_2_ molecules gather into nuclei growing up to nanoparticles of several to tens of nanometers in dimension in liquate Sn-0.4Ti matrix. These TiO_2_ nanoparticles adhere to the compact interfacial layer between the Sn-0.4Ti alloy and quartz glass. We hope that the results presented here will promote further works on Sn-Ti design to further improve the welding quality between the Sn-Ti alloy and quartz glass. Because almost all kinds of solders need to remove their oxide layer at welding, the results can also provide a feasible research idea to remove any oxide layer on the surface of solders, especially for similar active solder for different kinds of glass or ceramic sealing.
